# No Impact of Vancomycin MIC, AUC, or AUC/MIC in *Enterococcus faecium* Bacteremia

**DOI:** 10.1111/fcp.70039

**Published:** 2025-08-13

**Authors:** Anne Limelette, Thibaut Tromeur, Rami Rhaiem, Morgane Bonnet, Yohan N'Guyen

**Affiliations:** ^1^ Laboratoire de Bactériologie Pôle de Biologie Reims France; ^2^ Service de Médecine Interne, Maladies Infectieuses et Immunologie Clinique Hôpital Robert Debré Reims France; ^3^ Service de Chirurgie Générale, Digestive et Endocrinienne Hôpital Robert Debré Reims France; ^4^ Pharmacie Hospitalière Hôpital Robert Debré Reims France; ^5^ Consultations de Médecine Interne, Maladies Infectieuses et Immunologie Clinique Hôpital Robert Debré Reims France; ^6^ UMR‐S 1320 CardioVir. Université de Reims Champagne Ardenne Reims France

**Keywords:** AUC, bacteremia, *Enterococcus Faecium*, MIC, PK/PD, vancomycin

## Abstract

**Background:**

There is no clear pharmacokinetic and pharmacodynamic (PK/PD) target during vancomycin‐susceptible *Enterococcus faecium* bacteremia (EFB).

**Objectives:**

To investigate whether in‐hospital mortality was associated with susceptibility to amoxicillin or vancomycin minimum inhibitory concentration (MIC) of the strain and with area under the curve over 24 h (AUC) and AUC/MIC during EFBs.

**Methods:**

All *E. faecium* strains isolated from blood cultures performed between January 1, 2017, and December 31, 2022, were included, and clinicobiological data were retrospectively extracted from corresponding medical records. The Vancomycin MICs were estimated using the VITEK 2 automated system. AUC was calculated among patients who received vancomycin during their first episode of EFB with available data.

**Results:**

Two hundred fifteen *E. faecium* strains not susceptible to amoxicillin had been isolated in 207 patients (125 male, median age 69 [1–98] years) with biliary and digestive tract diseases, hematologic malignancies, or COVID‐19 in 124 (59.9%), 35 (16.9%), and 17 (8.2%) cases, respectively. The median vancomycin MIC was 0.5 [0.5–2] mg/L, and 67 patients (32.3%) died during the hospitalization. In‐hospital mortality was not associated with susceptibility to amoxicillin (*p* = 0.14) or vancomycin MIC (*p* = 0.07) of the strain. Neither mean AUC (592.7 versus 521.7mgh/L) nor mean AUC/MIC ratio (1066.5 versus 1000.5) was associated with in‐hospital mortality (*p* = 0.17 and *p* = 0.54, respectively).

**Conclusions:**

Besides amoxicillin susceptibility and vancomycin MIC of the strain, there was no significant association between in‐hospital mortality and vancomycin AUC or AUC/MIC. Retrospective observational studies focusing on in‐hospital mortality among patients with severe comorbidities may not be adequate for the determination of the PK/PD target of vancomycin.

**Abbreviations**
AUC: area under the curve over 24 h
MIC: minimum inhibitory concentration
PK/PD: pharmacokinetic and pharmacodynamic
MRSA: methicillin‐resistant *Staphylococcus aureus*

EFB:
*E. faecium* bacteremia
CKDEPI: Chronic Kidney Disease Epidemiology Collaboration
mAUC0‐24: mean AUC over 24 h
mAUC0‐24/MIC: mean AUC over 24 h to MIC ratio
mAUC0‐24cont: mean AUCs over 24 h for continuous infusion
mAUC0‐24cont/MIC: mean AUCs over 24 h for continuous infusion to MIC ratio
mAUC0‐24dis: mean AUCs over 24 h for discontinuous infusion
mAUC0‐24 dis/MIC: mean AUCs over 24 h for discontinuous infusion to MIC ratio
mAUC0‐24d1‐3: mean AUC over 24 h during the first 72 h following EFB
mAUC0‐24d1‐3/MIC: mean AUC over 24 h during the first 72 h following EFB to MIC ratio

## 1 Introduction

Obtaining a ratio of area under the curve over 24 h (AUC) to minimum inhibitory concentration (MIC) of 400 to 600 is now the recommended pharmacokinetic and pharmacodynamic (PK/PD) target when using vancomycin during methicillin‐resistant *S. aureus* (MRSA) infections [1].

However, there is no such specific target concerning *Enterococcus* spp. bacteremia because the few data from literature focusing on vancomycin susceptible *Enterococcus* spp. strains give discrepant results [2, 3]. Indeed, a Korean study [2] suggested that a trough concentration of vancomycin lower than 15 mg/L was associated with 1‐month mortality whereas another one [3] suggested that a trough concentration of vancomycin higher than 13.9 mg/L was also associated with 1‐month mortality. None of these two retrospective studies [2, 3] found an association of vancomycin AUC/MIC ratio with mortality. Both studies [2, 3] included *E. faecium* strains but also *Enterococcus faecalis* strains which are more likely susceptible to ampicillin and levofloxacin [4]. This could have led to a potential bias in case of concurrent antibiotic use, when evaluating the impact of vancomycin exposure on mortality, suggesting that vancomycin exposure did not reflect fully the infection‐attributable mortality [3]. Moreover, two studies focusing on all *Enterococcus* spp. strains showed an association between vancomycin AUC/MIC>389–400 and outcomes [5, 6], another study focusing mainly on *E. faecium* strains showed an association between vancomycin AUC > 420mgh/L (but not AUC/MIC) and mortality [7] whereas a last one focusing on *E. faecium* strains did not find any association between vancomycin AUC/MIC or trough concentrations and mortality [8]. In current practice, some physicians use daptomycin instead of vancomycin, even in case of Vancomycin susceptible *E. faecium* bacteremia (EFB).

In our tertiary center, vancomycin‐resistant *E. faecium* strains were not endemic, and the vancomycin‐susceptible *E. faecium* strains frequently harbored resistance to Amoxicillin and Levofloxacin. As quasi‐experimental studies have been carried out in the past when no therapeutic alternative was available [9], we wished to study the potential impact of vancomycin AUC/MIC ratio on the outcome of EFB in our center, in order to determine if vancomycin AUC/MIC ratio was really relevant for EFB.

In the present study, we sought first (i) to investigate whether in‐hospital mortality was not associated with the strain's susceptibility to amoxicillin and levofloxacin and (ii) to compare the MICs of vancomycin and daptomycin of available strains and to investigate the association between vancomycin MICs and the outcome of EFBs, before we began (iii) to investigate the impact of vancomycin AUC and AUC/MIC on the outcome of EFBs.

## 2 Material and Methods

### 2.1 Bacterial Strains, Study Population, and Ethics

**FIGURE 1:**
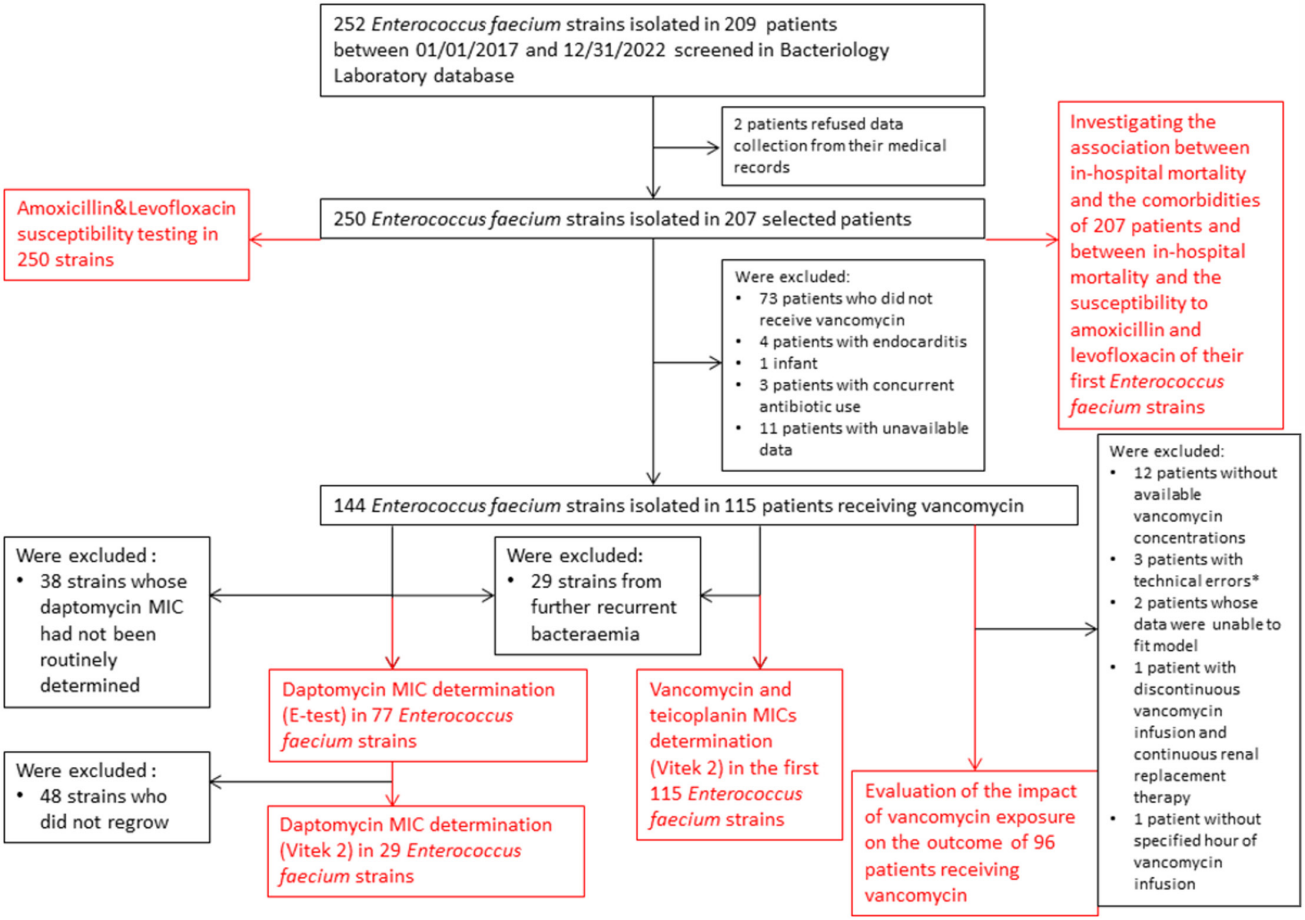
Flowchart of the study's patients and *Enterococcus faecium* strains. The study's objectives are noticed in red. MIC, minimum inhibitory concentration. *Technical errors: stopping vancomycin perfusion while awaiting the result of serum concentration (*n* = 1), wrong vancomycin infusion flow rate (*n* = 1), and aberrant vancomycin serum concentration with sample obtained near the site of infusion (*n* = 1).

All *E. faecium* strains isolated from blood cultures performed in Reims University Hospital between January 1, 2017, and December 31, 2022 were extracted from the Bacteriology Laboratory database (Labo Serveur, Inlog). Corresponding patient identities had also been extracted, and letters informing that data would be collected from medical records for research purposes were sent to the patients who were still alive, on February 17, 2023, in accordance with French legislation. Two patients refused the collection of data from their medical records. Among previously deceased patients, none had previously objected to the further use of their medical data during their hospitalization before they died. Thus, 250 *E. faecium* strains from 208 patients were included in the present study (Figure 1). After March 16, 2023, retrospective demographical, clinical, and biological data were extracted from medical records before anonymization. The reported weight, height, or creatinine values were the nearest values from the date of the first *E. faecium* bacteremia (EFB). Creatinine clearance values were calculated using the Chronic Kidney Disease Epidemiology Collaboration (CKDEPI) method [10]. Maximum values were capped at 90 mL/min. In case of intermittent hemodialysis, the interdialysis creatinine clearance was arbitrarily set at 10 mL/min. Data confidentiality was preserved throughout this internal study (Reims University Hospital GDPR register number MR00416022023), in accordance with the principles of the Declaration of Helsinki.

### 2.2 Evaluating the Outcome of *E. faecium* Bacteremia

The variables of interest were the in‐hospital mortality defined as all‐cause mortality during the hospitalization in which the bacteremia occurred and the recurrence of bacteremia defined as any new blood culture positive for any *E. faecium* strain occurring more than three calendar days and less than 1 year after the first EFB.

### 2.3 Antibiotic Susceptibility Testing and Minimum Inhibitory Concentration Determination

Amoxicillin and levofloxacin but also linezolid and vancomycin susceptibility testing had been routinely performed for all the 250 strains collected between January 1, 2017, and December 31, 2022, using the VITEK 2 automated system (BioMerieux, Marcy l'Etoile, France) and according to each year's CASFM/EUCAST guidelines [11]. When the susceptibilities to amoxicillin or levofloxacin were expressed among the 207 patients (and not the 250 strains), only the first strain involved was considered.

The vancomycin and teicoplanin minimum inhibitory concentrations (MICs) had been determined using the VITEK 2 automated system and cards AST‐P667 (BioMerieux, Marcy l'Etoile, France) according to the manufacturer's instructions, for each first *E. faecium* strain isolated in the blood of 115 patients who received vancomycin (i) without concurrent antibiotic use or endocarditis and (ii) with available data (Figure 1). The daptomycin MICs had been routinely estimated using a commercially available E‐test (E‐test Vancomycine, BioMerieux, Marcy l'Etoile, France) for 77 strains. To obtain a daptomycin MIC value with a method allowing comparison with that of vancomycin, daptomycin MICs were also estimated using the VITEK 2 automated system (BioMerieux, Marcy l'Etoile, France) by forcing the species as “ *E. faecalis* ” for 29 strains with effective regrowth (Figure 1). The values determined by VITEK 2 as inferior to 0.5 mg/L (< 0.5 mg/L) for vancomycin and as inferior to 0.12 mg/L (< 0.12 mg/L) for daptomycin were capped at 0.5 mg/L and 0.12 mg/L, respectively.

### 2.4 Evaluating the Vancomycin Area Under the Curve Over 24 h to Minimum Inhibitory Concentration Ratio

Vancomycin exposure was estimated by calculating the area under the curve over 24 h (AUC) each time a serum vancomycin concentration value was available among patients who received vancomycin during their first episode of EFB. The data associated with subsequent EFB in the same patient previously treated by vancomycin were excluded from this analysis (Figure 1) as were the patients who experienced infective endocarditis or the neonates/infants because of probable different PK/PD targets and extreme PK variability, respectively, compared to the rest of the study population. The patients in which the AUC could not be calculated or estimated (absence of prescription of vancomycin, absence of data concerning vancomycin concentration or infusion, and data unable to fit model) and those with susceptible *E. faecium* strains, who received simultaneous treatment by beta‐lactam such as amoxicillin, levofloxacin, or linezolid, were also excluded from this analysis (Figure 1). A prior treatment by daptomycin or vancomycin was not an exclusion criterion. The vancomycin AUCs were no more estimated with serum vancomycin concentrations sampled once the treatment by vancomycin was definitively withdrawn.

- In case of continuous vancomycin infusion, AUC was calculated by multiplying serum vancomycin concentration by a factor of 24 [1].
- In case of discontinuous vancomycin infusion, AUC was estimated by Bayesian model estimation using the free VancoCalc software [12, 13]. The creatinine blood value was the nearest creatinine blood value from the date of EFB, except when vancomycin treatment was anterior to EFB. In that case, the creatinine blood value was the nearest from the beginning of vancomycin treatment.
  ○ In case of discontinuous vancomycin infusion and intermittent hemodialysis, the AUC was calculated only for the vancomycin serum concentration value before the first hemodialysis following EFB.
  ○ In case of discontinuous vancomycin infusion and continuous renal replacement therapy, AUCs could not have been correctly estimated, and the patient was excluded from this analysis (Figure 1).

A vancomycin arithmetical mean AUC over 24 h (mAUC0‐24) was calculated for each patient experiencing EFB and receiving vancomycin using estimated and calculated AUCs (see above). A vancomycin mean AUC over 24 h to MIC ratio (mAUC0‐24/MIC) was calculated for each patient by dividing this mean AUC value by the vancomycin MIC determined using VITEK 2 automated system (BioMerieux, Marcy l'Etoile, France) (see above). In the same way, vancomycin arithmetical mean AUCs over 24 h were calculated for each patient, using AUC values obtained for continuous infusion (mAUC0‐24cont), for discontinuous infusion (mAUC0‐24dis) and during the first 72 h following EFB (mAUC0‐24d1‐3) respectively. mAUC0‐24d1‐3 was set at 0 when patients received vancomycin more than three calendar days after EFB. All these values were also divided by the vancomycin MIC to obtain vancomycin mean AUC over 24 h to MIC ratios for continuous infusion (mAUC0‐24cont/MIC), discontinuous infusion (mAUC0‐24dis/MIC) and the first 72 h (mAUC0‐24d1‐3/MIC) for each patient.

### 2.5 Statistical Analysis

Quantitative variables usually expressed as median + range were compared using the Mann–Whitney *U* test and qualitative variables expressed as percentages were compared using Fischer's exact test or Pearson's chi‐square test if applicable. A linear regression was performed to study the correlation between non‐normally distributed quantitative variables. A *p* value < 0.05 was considered as significant. When a logistic regression analysis was performed, only variables with a *p* value ≤ 0.10 in univariate analysis were entered in multivariate logistic regression model. Statistical analyses were performed using EpiInfo 7.2.2.6 (CDC, Atlanta, Georgia, United States), GraphPad Prism (GraphPad Software, San Diego California, United States) and MedCalc version 22.007 (MedCalc Software Ltd., Ostend, Belgium; https://www.medcalc.org; 2023) softwares.

## 3 Results

### 3.1 Investigating the Association Between In‐Hospital Mortality and the Susceptibilities of *E. faecium* Strains to Amoxicillin and Levofloxacin

**FIGURE 2:**
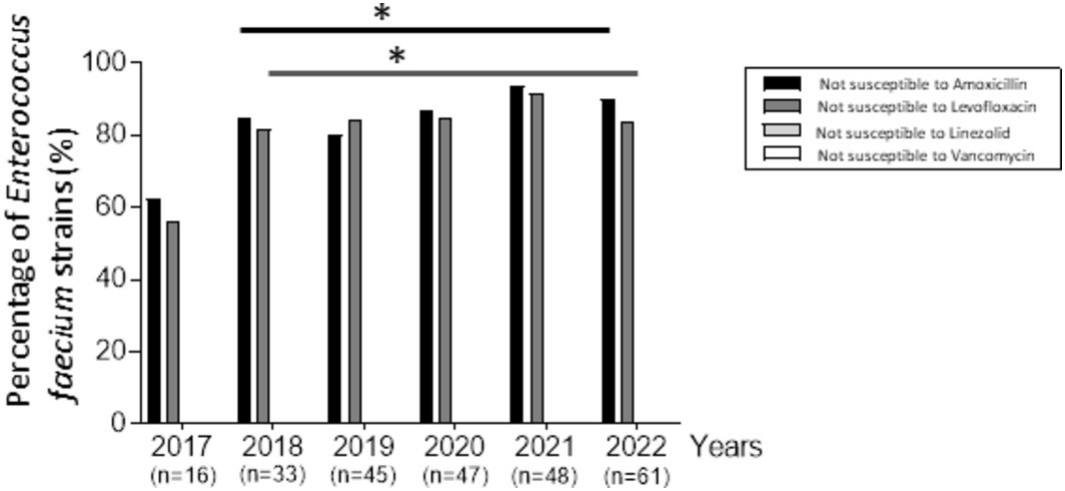
Percentage of *Enterococcus faecium* strains not susceptible to amoxicillin, levofloxacin, linezolid, and vancomycin across years. *p > 0.1 according to Fischer's exact test, when comparing the percentage of strains not susceptible to amoxicillin (black horizontal bar) or not susceptible to levofloxacin (dark gray horizontal bar) across years.

Among the 250 strains included, 215 (86.0%) and 209 (83.6%) *E. faecium* strains were not susceptible to amoxicillin and levofloxacin, respectively. All were susceptible to vancomycin or linezolid. The percentage of *E. faecium* strains not susceptible to amoxicillin and levofloxacin remains stable across the years, without significant difference (*p* > 0.1) except between 2017 and the rest of the study period (Figure 2).

These 250 strains had been isolated in 207 patients (125 male, median age 69 [1–98] years) with biliary and digestive tract diseases, hematologic malignancies, or while hospitalized for COVID‐19 in 124 (59.9%), 35 (16.9%), and 17 (8.2%) cases, respectively (Table 1). The details of biliary and digestive tract diseases are given in Table S1. Among these 207 patients, 116 (56.0%) were deceased at the date of February 17, 2023, but only 67 (32.3%) died during the hospitalization where EFB occurred (Table 1).

**TABLE 1:** Association between in‐hospital mortality and the study patient's comorbidities or *Enterococcus faecium* strains susceptibility to amoxicillin and levofloxacin.

Characteristics	All patients, *n* = 207	Patients who died during hospitalization where EFB occurred, *n* = 67	Survivors of the hospitalization where EFB occurred, *n* = 136^a^	
Male sex (*n*) (%)	125 (60.3)	41 (61.1)	81 (59.5)	0.82
Median age (years) [range]	69 [1–98]	70 [46–96]	69 [1–96]	0.10
Patients with biliary and digestive tract diseases (*n*) (%)	124 (59.9)	42 (62.6)	80 (58.8)	0.87
Patients with any malignant neoplasm^b^ (*n*) (%)	112 (54.1)	33 (49.2)	77 (56.6)	0.19
Patients with solid malignant neoplasm (*n*) (%)	77 (37.1)	26 (38.8)	49 (36.0)	0.88
Patients with hematologic malignancies (*n*) (%)	36 (17.3)	7 (10.4)	29 (21.3)	0.04
Patients hospitalized for COVID‐19 (*n*) (%)	17 (8.2)	12 (17.9)	5 (3.6)	0.0008
Patients admitted to intensive care unit (*n*) (%)	82 (39.6)	38 (56.7)	43 (31.6)	0.001
Bacteremia with *E. faecium* strain^c^ not susceptible to amoxicillin (*n*) (%)	175 (84.5)	62 (92.5)	109 (80.1)	0.02
Bacteraemia with *E. faecium* strain^c^ not susceptible to levofloxacin (*n*) (%)	170 (82.1)	59 (88.0)	107 (78.6)	0.10

^a^ Missing data = 4.

^b^ One patient had both solid malignant neoplasm and hematologic malignancy.

^c^ First strain in case of recurrent bacteremia.

The in‐hospital mortality was significantly higher among the patients who were admitted to the intensive care unit (whatever the reason) or for COVID‐19 infection than among those who were not. The in‐hospital mortality was significantly lower in patients who had hematologic malignancies, as it was not observed among patients with biliary or digestive and other neoplastic diseases. The in‐hospital mortality was significantly higher among patients whose *Enterococcus faecium* strains were not susceptible to Amoxicillin (but not levofloxacin) (Table 1). The results of multivariate logistic regression analysis are shown in Table 2. Only age, admission to the intensive care unit, and COVID‐19 were independently associated with in‐hospital mortality, but the susceptibility to amoxicillin of the strain was not.

**TABLE 2:** Factors independently associated with in‐hospital mortality during *Enterococcus faecium* bacteremia.

Characteristics	Odds radio	Confidence interval 95%	*p*
Age (years)	1.03	[1.006–1.06]	0.01
Hematologic malignancy	0.50	[0.19–1.32]	0.16
Hospitalization for COVID‐19	3.44	[1.07–11.07]	0.03
Admission to intensive care	2.41	[1.21–4.81]	0.01
Strain not susceptible to amoxicillin	2.77	[0.71–10.83]	0.14
Strain not susceptible to levofloxacin	0.95	[0.29–3.08]	0.93

### 3.2 Vancomycin, Teicoplanin, and Daptomycin Minimum Inhibitory Concentrations Among the Available *E. faecium* Strains Isolated in Patients Who Received Vancomycin

**FIGURE 3:**
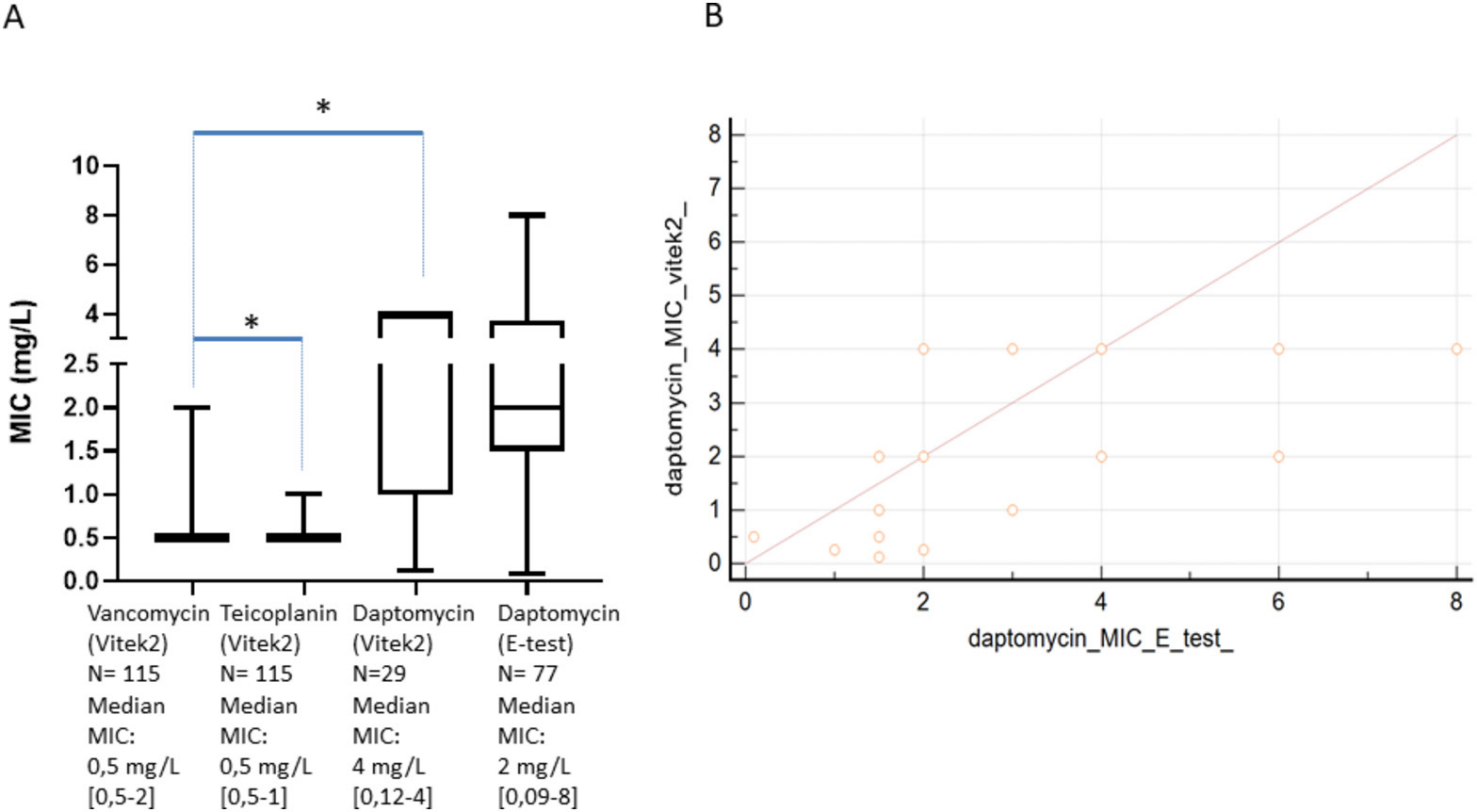
Vancomycin, teicoplanin, and daptomycin minimal inhibitory concentrations (MICs) among the available *Enterococcus faecium* strains isolated in the blood of the patients who received vancomycin. (A) Boxplots comparing vancomycin, teicoplanin, and daptomycin minimal inhibitory concentrations in available strains. *p < 0.05 according to the Mann–Whitney *U* test. (B) Scatter diagram of daptomycin MICs according to its determination assay (E‐Test or Vitek2).

Vancomycin, teicoplanin MICs were determined using VITEK 2 for the 115 first *E. faecium* strains of the 115 adult patients who received vancomycin (i) without concurrent antibiotic use, (ii) with available data, and (iii) without endocarditis (Figure 1). The daptomycin MICs had been determined using E‐test for 77 *E. faecium* strains and also using VITEK 2 for a sample of 29 out of these 77 strains. The vancomycin, teicoplanin, and daptomycin MICs are shown in Figure 3.

The teicoplanin MICs were statistically lower (*p* = 0.003) than the vancomycin MICs, which were lower than the daptomycin MICs determined using Vitek 2 (*p* < 0.0001). The linear regression between daptomycin MIC determined by Vitek 2 and that determined by E‐test gave a poor coefficient of determination (*R* ^2^ = 0.29).

### 3.3 Evaluating the Impact of Vancomycin Minimum Inhibitory Concentrations on the Outcome of *E. faecium* Bacteremia

Clinical and demographic data from the 115 patients who received vancomycin (i) without concurrent antibiotic use, (ii) with available data, and (iii) without endocarditis (Figure 1) are given in Table 3.

**TABLE 3:** Characteristics of the 115 study adult patients with available data who experienced *Enterococcus faecium* bacteremia without endocarditis and who received vancomycin without concurrent antibiotic use.

Characteristics	Aforementioned study patients, *n* = 115
Male sex (*n*) (%)	67 (58.2)
Median age (years) [range]	68 [33–94]
Median body weight (kg) [range]	70 [31–150]
Median height (cm) [range]	167 [148–191]
Median body mass index (kg/cm^2^) [range]	24.8 [10.6–58.5]
Median creatinine value at the time of bacteremia (μmol/L) [range]	75 [19–649]
Median creatinine clearance (ml/min) [range]	81 [8–90]
Biliary and digestive tract diseases (*n*) (%)	69 (60.0)
Malignant neoplasm (*n*) (%)	65 (56.5)
Hematologic malignancies (*n*) (%)	23 (20.0)
Hospitalization for COVID‐19 (*n*) (%)	14 (12.1)
Bacteremia with *E. faecium* strain not susceptible to amoxicillin (*n*) (%)	107 (93.0)
Bacteremia with *E. faecium* strain not susceptible to levofloxacin (*n*) (%)	104 (90.4)
Admission to intensive care unit (*n*) (%)	56 (48.6)
Deceased at February 17, 2023 (*n*) (%)	67 (58.2)
In‐hospital mortality (*n*) (%)	40 (34.7)
Bacteremia recurrence (*n*) (%)	18 (15.6)

Concerning the outcome of EFB, in‐hospital mortality was 34.7% (40 patients) and 18 patients (15.6%) experienced recurrence of EFB (Table 3). The median time delay between first EFB and recurrence was 16.5 [4–261] days.

There was no statistically significant difference between the vancomycin MICs of patients with in‐hospital mortality or EFB recurrence and those of patients without (*p* = 0.07 or *p* = 0.81, respectively).

### 3.4 Evaluating the Impact of Vancomycin Area Under the Curve Over 24 h to Minimum Inhibitory Concentration Ratio on the Outcome of *E. faecium* Bacteremia

The data of 96 patients were analyzed; 19 patients had been excluded, among those 12 because no serum vancomycin concentrations were available (Figure 1).

The vancomycin treatment had been started before the onset of EFB in two patients (2.1%) and a prior treatment by daptomycin had been given before vancomycin in four patients (4.2%).

Among these 96 patients, vancomycin was administrated by continuous and intermittent infusion in 79 and 28 cases, respectively, some patients receiving both types of infusion. The median length of continuously infused vancomycin treatment was 7 days [0–46] among the 96 patients. The median number of infusions was 7 [1–79] among the 28 patients who received intermittent vancomycin infusions. The median numbers of serum vancomycin concentrations obtained over a time period up to 61 days were 3 [0–18] and 2 [0–23] among the patients who received continuous and intermittent vancomycin infusions, respectively. The vancomycin treatment was started more than 3 calendar days after the onset of EFB in one case, giving mAUC0‐24d1‐3 values of 0.

Median mAUC0‐24 value was 534 [112–1592] mgh/L among all the 96 patients who received vancomycin. Median values were 531.4 [112.8–1504.8] and 571 [270–1592.1] mgh/L for patients who received continuous (mAUC0‐24cont) and discontinuous (mAUC0‐24dis) vancomycin infusions, respectively. Median mAUC0‐24d1‐ 3 value was 460 [0–516] mgh/L.

**FIGURE 4:**
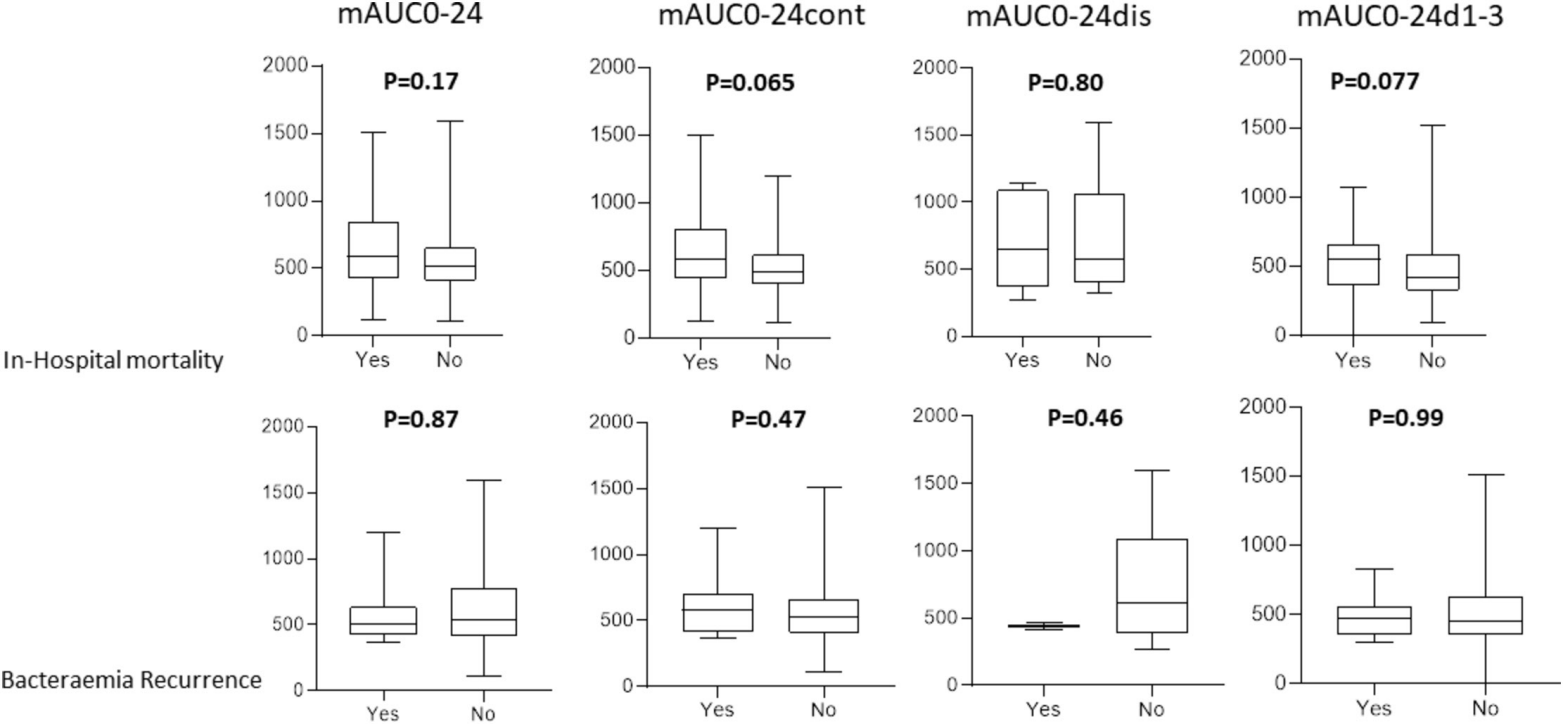
Boxplots comparing all vancomycin mean AUC (mAUC0‐24), continuously infused vancomycin mean AUC to MIC ratio (mAUC0‐24cont), discontinuously infused vancomycin mean AUC to MIC ratio (mAUC0‐24dis) and the first 72 h vancomycin mean AUC to MIC ratio (mAUC0‐24d1‐3) among patients who died during hospitalization where *Enterococcus* bacteremia occurred or who experienced *Enterococcus* bacteremia recurrence and among those who did not. In bold, *p* values obtained after comparison using Mann–Whitney *U* test.

**FIGURE 5:**
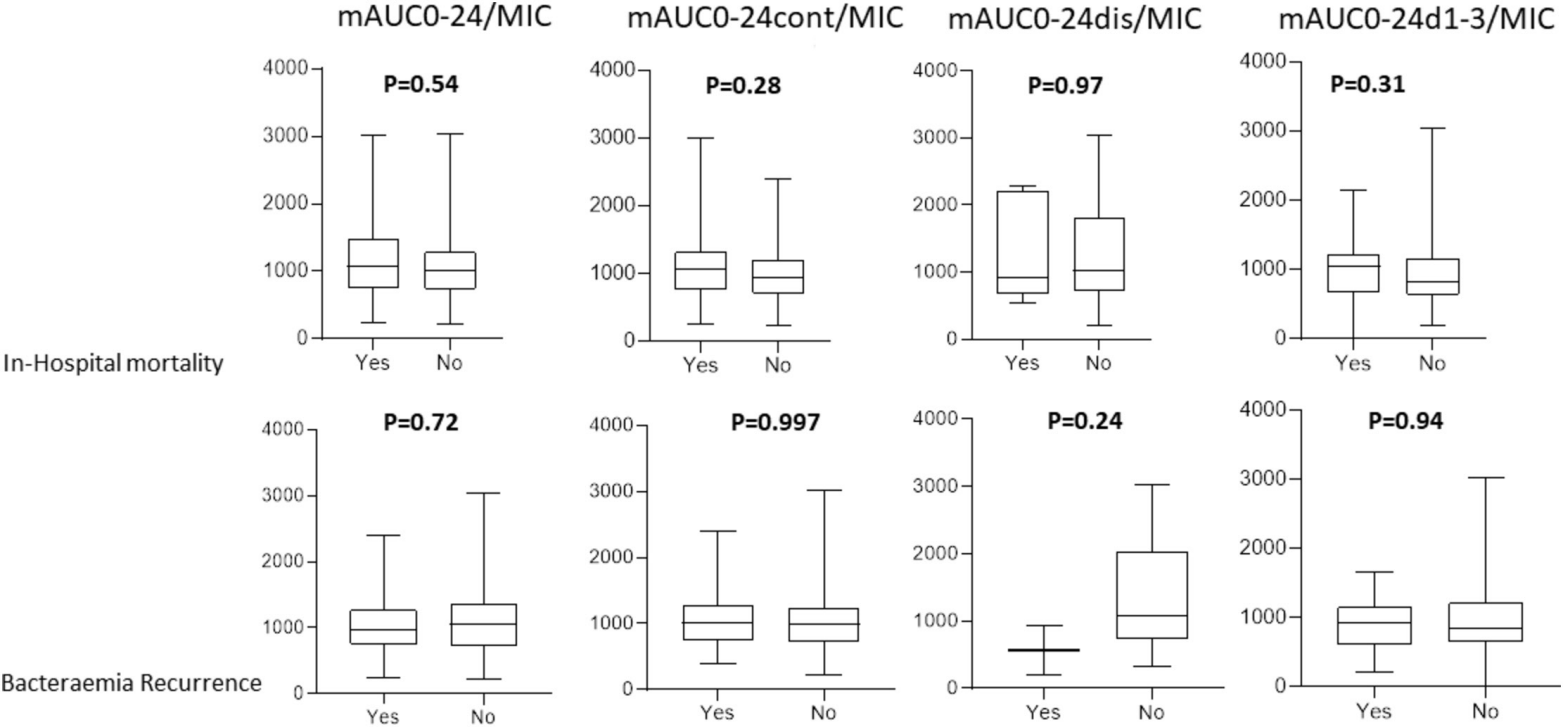
Boxplots comparing all vancomycin mean AUC to MIC ratio (mAUC0‐24/MIC), continuously infused vancomycin mean AUC to MIC ratio (mAUC0‐24cont/MIC), discontinuously infused vancomycin mean AUC to MIC ratio (mAUC0‐24dis/MIC) and the first 72 h vancomycin mean AUC to MIC ratio (mAUC0‐24d1‐3/MIC) among patients who died during hospitalization where *Enterococcus* bacteremia occurred or who experienced *Enterococcus* bacteremia recurrence and among those who did not. In bold, *p* values obtained after comparison using Mann–Whitney *U* test.

Among these 96 patients, 33 (34.3%) died during the hospitalization in which the bacteremia occurred, and 13 patients (13.5%) experienced recurrence of EFB. There was no significant association between in‐hospital mortality or recurrence and AUCs (all *p* > 0.05) (Figure 4). Median mAUC0‐24/MIC, mAUC0‐24cont/MIC, mAUC0‐24dis/MIC, or mAUC0‐24d1‐3/MIC values were not significantly lower either among the patients who died or experienced recurrence than among those who did not (all *p* > 0.05) (Figure 5).

There was no statistically significant difference in the occurrence of in‐hospital mortality or recurrence between patients with mAUC0‐24 or mAUC0‐24d1‐3 values below and above 400 mgh/L and between patients with mAUC0‐24/MIC or mAUC0‐24d1‐3/MIC values below and above 400 (all *p* > 0.10, data not shown).

## 4 Discussion

In the present report, we observed that neither amoxicillin nor levofloxacin susceptibilities of the strain, nor vancomycin MICs of the strain, nor estimated vancomycin AUC and AUC/MIC ratios were associated with in‐hospital mortality or recurrence during *E. faecium* bacteremia (EFB) and EFBs treated by vancomycin, whether it was administered continuously and within 72 h or not. Taken together, these results suggested that the PK/PD target of vancomycin (which should remain the drug of choice in light of MICs) remains unknown and that retrospective observational studies focusing on in‐hospital mortality among patients with severe comorbidities (Table 1, Table S1 and Table 3) may not be adequate for the determination of such PK/PD target of vancomycin during EFB. Interestingly, the Vancomycin Minimal Inhibitory concentrations (MICs), determined using Vitek 2, were significantly lower than those of daptomycin among a subset of available strains sampled in the blood of patients who received vancomycin.

The main limitations of this study are as follows: (i) its retrospective and observational nature, leading to unavailable data especially concerning serum vancomycin concentrations, non‐routinely performed daptomycin MICs, or absence of infective endocarditis according to echocardiography; (ii) the absence of regrowth of two thirds (48 out of 77) of *Enterococcus Faecium* strains whose daptomycin MICs have been determined; (iii) the determination of daptomycin MICs using the Vitek2 system, which has not been validated by the manufacturer for *E. faecium* strains; (iv) the retrospective determination of vancomycin AUC using the available data reported on the medical records (dose, infusion rate and timing, sample timing, and concentration results) and the free VancoCalc software, which is based on the PK model of Buelga et al. [14] established on a population of patients with hematological malignancies (underrepresented in this study, Table 1), with inter‐individual variability associated with clearance of 44% in this model; and (v) the absence of collected data concerning vancomycin nephrotoxicity in this study focusing on the AUCs and AUC/MIC ratios and their association with in‐hospital mortality or recurrence and not on the benefit/risk ratio of vancomycin.

As ours, three retrospective studies [2, 3, 8] involving less than 100 patients and another one [7] involving a bit more than 100 patients were not able to determine an association between mortality during enterococcal bacteremia and vancomycin AUC/MIC, which is the recommended PK/PD target during MRSA infections. Other discrepant PK/PD targets were evidenced by two of these studies [2, 3], but it would suggest that vancomycin may not have the same bactericidal behavior on *Enterococcus* spp. and *Staphylococcus* spp. strains, which are both gram‐positive cocci. Another explanation would be that these studies, as ours, were possibly not powered enough to show an association between 1‐month mortality and vancomycin AUC/MIC. Only the study of Jumah [5] evidenced such an association between 1‐month mortality and vancomycin AUC/MIC > 389 among *Enterococcus* spp. bacteremia. The outcome associated with a vancomycin AUC/MIC > 400, which was evaluated in the study of Katip and Oberdorfer, was not the same [6]. Interestingly, the study of Tangvichitrerk et al. [7] showed an association between 1‐month mortality and a vancomycin AUC > 420mgh/L (but not AUC/MIC ratio). Our study performed in France has not been able to show the associations with outcomes of these three studies performed in Asia [5, 6, 7]. The portal of entry of the enterococcal bacteremia in these three studies [5, 6, 7] seemed to be less likely an intra‐abdominal infection than it was in ours, suggesting the studies population may not be comparable. An association between overrepresentation of digestive streptococci in a definite kind of infection and fecal carriage or nutritional factors in some geographically restricted populations has been hypothesized elsewhere [15]. Lastly, the study of Nakakura et al. [7] focusing on EFB evidenced an association with Charlson Comorbidity Index and 1‐month mortality, assertion that we feel relevant in light of the data of our study (see below).

Even if the PK/PD target of vancomycin was not universally determined during EFB, the vancomycin MICs of *E. faecium* strains were significantly lower in our study than the daptomycin MICs. To be comparable with the values of vancomycin MICs, daptomycin MICs were estimated using the VITEK 2 automated system by forcing the species as “ *E. faecalis*.” This method has not been validated by the manufacturer, and the correlation with E‐test was poor for the subset of *E. faecium* strains, which had their Vancomycin MICs determined by both E‐test and VITEK 2 assays. However, the VITEK 2 assay seemed to give lower MIC values than E‐test (Figure 3B). Moreover, the vancomycin MICs could have been more overestimated than daptomycin MICs because the value was capped at 0.5 mg/L when the VITEK 2 system gave an estimation of MICs as inferior to 0.5 mg/L for vancomycin instead of 0.12 mg/L for daptomycin. Even if they were biased, these MIC data are fully in line with those reported in EUCAST ECOFFS [4]. This suggested that vancomycin (which is cheaper and more frequently used worldwide than teicoplanin) should remain the drug of choice for the treatment of bacteremia due to Amoxicillin and Levofloxacin resistant but Vancomycin susceptible *E. faecium* strains, which accounted for more than 80% of the *E. faecium* strains in our center.

Therefore, as the determination of the PK/PD target of vancomycin during EFB remains relevant, maybe other studies than retrospective monocentric observational studies focusing on mortality among patients with severe comorbidities shall be performed. As stated above, this kind of retrospective monocentric studies [2, 3, 7, 8] like ours may not be powered enough to evidence an association between vancomycin AUC/MIC and outcomes such as mortality. But, mortality may not per se be the outcome of choice for the evaluation of the efficiency of vancomycin in our context. Indeed, taking into account that more than one half of the patients with comorbidities we included (Table 1, Table S1, and Table 3) were deceased 6 weeks after the end of the 5‐year study period, we tried to choose in‐hospital mortality instead of one‐month mortality as a proxy of the infection‐attributable mortality. Even with in‐hospital mortality, we did not evidence an association between amoxicillin and levofloxacin susceptibilities or vancomycin MICs of the strain as well as vancomycin AUC/MIC or AUC and mortality. The factors independently associated with in‐hospital mortality were age and admission to the intensive care unit (as described in almost all studies focusing on mortality), but also the occurrence of COVID‐19. In these cases, we presumed that the mortality corresponded more to the spontaneous mortality of a patient with pre‐Omicron COVID‐19 requiring prolonged invasive mechanical ventilation and experiencing multiple ventilator‐associated pneumonias than that of the EFB itself. Even with in‐hospital mortality, the patients seemed to die more of their comorbidities [8] (see above) and of the treatment of their comorbidities (potentially leading to intensive care unit admission) than of the EFB itself. We therefore tried to investigate the recurrence of EFB as an alternative outcome, but there was no association with AUC/MIC either, maybe because of the low number of events of recurrence, as we defined it. Taken together, retrospective monocentric observational studies may not be adequate in our context for the determination of the PK/PD target of vancomycin during *E. faecium* infections. Instead of prospective multicentric studies with precise determination of vancomycin AUC/MIC ratio, maybe experimental studies should first be performed in our context, as it was for MRSA and vancomycin [16, 17].

## 5 Conclusion

The vancomycin area under the curve over 24 h to MIC ratio was not associated with in‐hospital mortality or recurrence of *E. faecium* bacteremia in our study. Retrospective observational studies focusing on in‐hospital mortality among patients with severe comorbidities may not be adequate in our context for the determination of the PK/PD target of vancomycin during EFB.

## Author Contributions

Yohan N'Guyen contributed to the study conception and design. Anne Limelette and Yohan N'Guyen extracted data. Rami Rhaiem and Morgane Bonnet took care of the patients. Thibaut Tromeur, Rami Rhaiem, and Yohan N'Guyen analyzed data. Thibaut Tromeur and Yohan N'Guyen wrote the first draft of the manuscript that was critically reviewed by all co‐authors.

## Conflicts of Interest

The authors declare no conflicts of interest.

## Transparency Declarations

None to declare.

## Supporting information


**TABLE S1** details of biliary and digestive tract diseases among the 124 patients with biliary and digestive tract disease who experienced *E. faecium* bacteraemia. Some patients could have more than one of the following:
