# Supplementary material for: No Impact of Vancomycin MIC, AUC, or AUC/MIC in Enterococcus faecium Bacteremia
Source: Fundam Clin Pharmacol. 2025 Aug 13;39(5):e70039. doi: 10.1111/fcp.70039 (PMC12351152; doi:10.1111/fcp.70039)
Supplement: Supplementary file 1 — TABLE S1 details of biliary and digestive tract diseases among the 124 patients with biliary and digestive tract disease who experienced E. faecium bacteraemia. Some patients could have more than one of the following: [file FCP-39-0-s001.docx]

Supplementary Table 1: details of biliary and digestive tract diseases among the 124 patients with biliary and digestive tract disease who experienced *Enterococcus faecium* bacteraemia. Some patients could have more than one of the following:

| Biliary infections : cholecystitis, cholangitis and biliary lithiasis | N=37 |
| --- | --- |
| Intraductal papillary mucinous neoplasm and pancreatic adenocarcinoma | N=26 |
| Cholangiocarcinoma | N=13 |
| Colorectal cancer and dysplasia | N=10 |
| Acute pancreatitis | N=9 |
| Peritoneal carcinomatosis and pseudomyxomea peritonei | N=8 |
| Acute alcoholic hepatitis, cirrhosis and hepatocellular carcinoma | N=5 |
| Gastrointestinal fistula | N=5 |
| Small Bowel adenocarcinoma | N=3 |
| Peritonitis | N=4 |
| Diverticulitis | N=4 |
| Gastric adenocarcinoma | N=3 |
| Neuroendocrine tumor | N=3 |
| Gastrointestinal bleeding without investigation | N=3 |
| Gastrointestinal Stromal tumor | N=2 |
| Mesenteric Ischaemia | N=2 |
| Colitis | N=2 |
| Enteritis | N=1 |
| Peptic strictures of the esophagus | N=1 |
| Ampullary Tumor | N=1 |
| Surgical and interventional complications* | N=8 |
| Miscellaneous** | N=2 |

* angiodysplasia electroagulation (n=2), post volvulus colo-colic anastomosis, small intestinal anastomotic leak , right hepatectomy, hemobilia post cholecystectomy, post aorta-splenic bypass, disconnected pancreatic duct syndrome.

** mesenteric lymphoma, hepatic rhabdomyosarcoma.
